# Supplementary material for: Containment of Fusarium culmorum and Its Mycotoxins in Various Biological Systems by Antagonistic Trichoderma and Clonostachys Strains
Source: J Fungi (Basel). 2023 Feb 22;9(3):289. doi: 10.3390/jof9030289 (PMC10056746; doi:10.3390/jof9030289)
Supplement: Supplementary file 1 [file jof-09-00289-s001.zip › SM - Tables S1-S12.pdf]

Table S1. List of *Trichoderma* and *Clonostachys* strains used in study.

**Culture code/CBS[a] no.**	**Origin**		**NCBI GenBank Assession no.[b]**	
	**Locality**	**Source**	**ITS**	***tef 1***
T. atroviride				
AN35/CBS136452	Central Poland	maize kernels	HQ292787	HQ292953
AN152/ CBS136453	Central Poland	triticale kernel	HQ292792	HQ292957
AN182/CBS136454	Central Poland	forest wood	HQ292794	HQ292965
AN206/CBS136455	Central Poland	mushroom compost	HQ292804	HQ292960
AN240	Karkonosze Mts, SP	forest wood	JX184119	JX184096
AN497	Gorce Mts, SP	forest wood	JX184119	JX184096
T. citrinoviride				
AN262	Central Poland	forest wood	HQ292847	-
AN393	Gorce Mts, SP	forest wood	JX184109	JX184086
T. cremeum				
AN392	Gorce Mts, SP	forest wood	JX184117	JX184094
T. hamatum				
AN120	Central Poland	forest wood	HQ292855	-
AN277	Central Poland	forest wood	HQ292857	-
T. harzianum				
AN150	Central Poland	forest wood	HQ292878	-
AN278	Central Poland	forest wood	HQ292890	-
AN360	Tatra Mts, SP	forest wood	JX184113	JX184090
T. koningiopsis				
AN143	Central Poland	forest wood	HQ292929	HQ292992
AN251	Karkonosze Mts, SP	forest wood	HQ292939	HQ292993
T. longibrachiatum				
AN197	Eastern Poland	mashroom factory	HQ292780	-
AN213	Central Poland	mashroom compost	HQ292781	-
T. longipile				
AN359	Tatra Mts, SP	forest wood	JX184115	JX184091
T. viride				
AN255	Karkonosze Mts, SP	forest wood	JX184121	JX184098
AN401	Gorce Mts, SP	forest wood	JX184122	JX184099
AN430	Central Poland	forest wood	HQ292926	HQ293014
AN826	Karkonosze Mts, SP	forest wood	JX184122	JX184099
T. viridescens				
AN323	Tatra Mts, SP	forest wood	JX184127	JX184103
AN405/ CBS136460	Gorce Mts, SP	forest wood	JX184127	JX184103
C. rosea* f. *rosea				
AN272	Western Poland	forest wood	-	-
AN291	Eastern Poland	soya	-	-
AN295	Eastern Poland	soya	-	-
C. rosea* f. *catenulata			-	-
AN297	Western Poland	soil	-	-
AN298	Eastern Poland	beans	-	-
AN300	Eastern Poland	*Betula pendula* leaf	-	-

Mts - mountains
SP - South Poland

Table S2a. Estimated *F. culmorum* KF 846 mycelial growth inhibition (%) by *Trichoderma* and *Clonostachys* strains during 7 days of co–incubation on PDA medium. Inhibition shown as % reduction relative to control.

**Species**	**Strain**	***Fusarium culmorum* KF 846 mycelial growth inhibition (%)**													
		**d1[a]**	**s.e.[b]**	**d2**	**s.e.**	**d3**	**s.e.**	**d4**	**s.e.**	**d5**	**s.e.**	**d6**	**s.e.**	**d7**	**s.e.**
C. rosea	AN272	46,67	5,554	-25	6,155	11,11	2,114	2,78	0,7096	17,65	0,9124	17,65	*	17,65	*
	AN291	10	6,593	-25	6,155	-11,11	2,362	-8,33	0,7501	23,53	0,8867	23,53	*	23,53	*
	AN295	10	6,593	-25	6,155	0	2,234	0	0,7195	16,47	0,9177	16,47	*	16,47	*
	AN297	36,67	5,801	0	5,438	11,11	2,114	0	0,7195	17,65	0,9124	15,29	*	15,29	*
	AN298	46,67	5,554	0	5,438	11,11	2,114	8,33	0,6902	15,69	0,9213	23,53	*	23,53	*
	AN300	46,67	5,554	-25	6,155	11,11	2,114	0	0,7195	17,65	0,9124	17,65	*	17,65	*
T. atroviride	AN152	66,67	5,166	35	4,586	44,44	1,807	50	0,5688	58,82	0,7617	58,82	*	58,82	*
	AN182	46,67	5,554	48,33	4,328	33,33	1,899	50	0,5688	58,82	0,7617	58,82	*	58,82	*
	AN206	60	5,278	15	5,047	11,11	2,114	33,33	0,6115	52,94	0,7784	52,94	*	52,94	*
	AN240	66,67	5,166	30	4,694	45,19	1,802	58,33	0,5512	70,59	0,7342	70,59	*	70,59	*
	AN497	66,67	5,166	48,33	4,328	44,44	1,807	40	0,5933	52,94	0,7784	52,94	*	52,94	*
T. citrinoviride	AN262	50	5,479	28,33	4,731	42,22	1,825	33,33	0,6115	52,94	0,7784	52,94	*	52,94	*
	AN393	50	5,479	40	4,484	20	2,023	33,33	0,6115	52,94	0,7784	52,94	*	52,94	*

T. cremeum	AN392	73,33	5,072	33,33	4,621	57,04	1,719	66,67	0,5363	64,71	0,7469	58,82	*	58,82	*
T. hamatum	AN120	33,33	5,89	38,33	4,517	11,11	2,114	33,33	0,6115	52,94	0,7784	52,94	*	52,94	*
	AN277	100	4,9	26,67	4,768	14,81	2,075	33,33	0,6115	52,94	0,7784	64,71	*	64,71	*
T. harzianum	AN150	70	5,116	50	4,299	31,85	1,912	40,56	0,5919	52,94	0,7784	52,94	*	52,94	*
	AN279	50	5,479	50	4,299	17,78	2,045	25	0,636	47,06	0,7969	52,94	*	52,94	*
	AN360	70	5,116	25	4,806	21,48	2,009	33,33	0,6115	52,94	0,7784	52,94	*	52,94	*
T. koningiopsis	AN143	40	5,715	20	4,924	34,07	1,892	50	0,5688	64,71	0,7469	64,71	*	64,71	*
	AN251	60	5,278	50	4,299	44,44	1,807	58,33	0,5512	70,59	0,7342	70,59	*	70,59	*
T. longibrachiatum	AN197	60	5,278	25	4,806	44,44	1,807	50	0,5688	64,71	0,7469	64,71	*	64,71	*
	AN213	80	4,997	21,67	4,884	42,22	1,825	50	0,5688	58,82	0,7617	58,82	*	58,82	*
T. longipile	AN359	70	5,116	46,67	4,358	47,41	1,785	50	0,5688	58,82	0,7617	58,82	*	58,82	*
T. viride	AN255	50	5,479	0	5,438	44,44	1,807	50	0,5688	64,71	0,7469	62,35	*	62,35	*
	AN401	100	4,9	75	3,963	22,22	2,001	33,33	0,6115	52,94	0,7784	58,82	*	58,82	*
	AN430	50	5,479	50	4,299	0	2,234	16,67	0,6623	64,71	0,7469	70,59	*	70,59	*
	AN826	80	4,997	48,33	4,328	45,93	1,796	58,33	0,5512	70,59	0,7342	64,71	*	64,71	*
T. viridescens	AN323	70	5,116	30	4,694	35,56	1,879	50	0,5688	64,71	0,7469	64,71	*	64,71	*
	AN405	70	5,116	25	4,806	41,48	1,83	50	0,5688	58,82	0,7617	58,82	*	58,82	*

[a]- Inhibition (%) on the day after inoculation
[b]- Standard error for three replicates
[c]- *Trichoderma* and *Clonostachys* strains (grey) showing the highest antagonistic ability against *F. culmorum* KF 846 on the 6th and 7th day of co-incubation

Table S2b. Visualisation of estimated *F. culmorum* KF 846 mycelial growth inhibition (%) by *Trichoderma* and *Clonostachys* strains during 7 days of co–incubation on PDA medium.

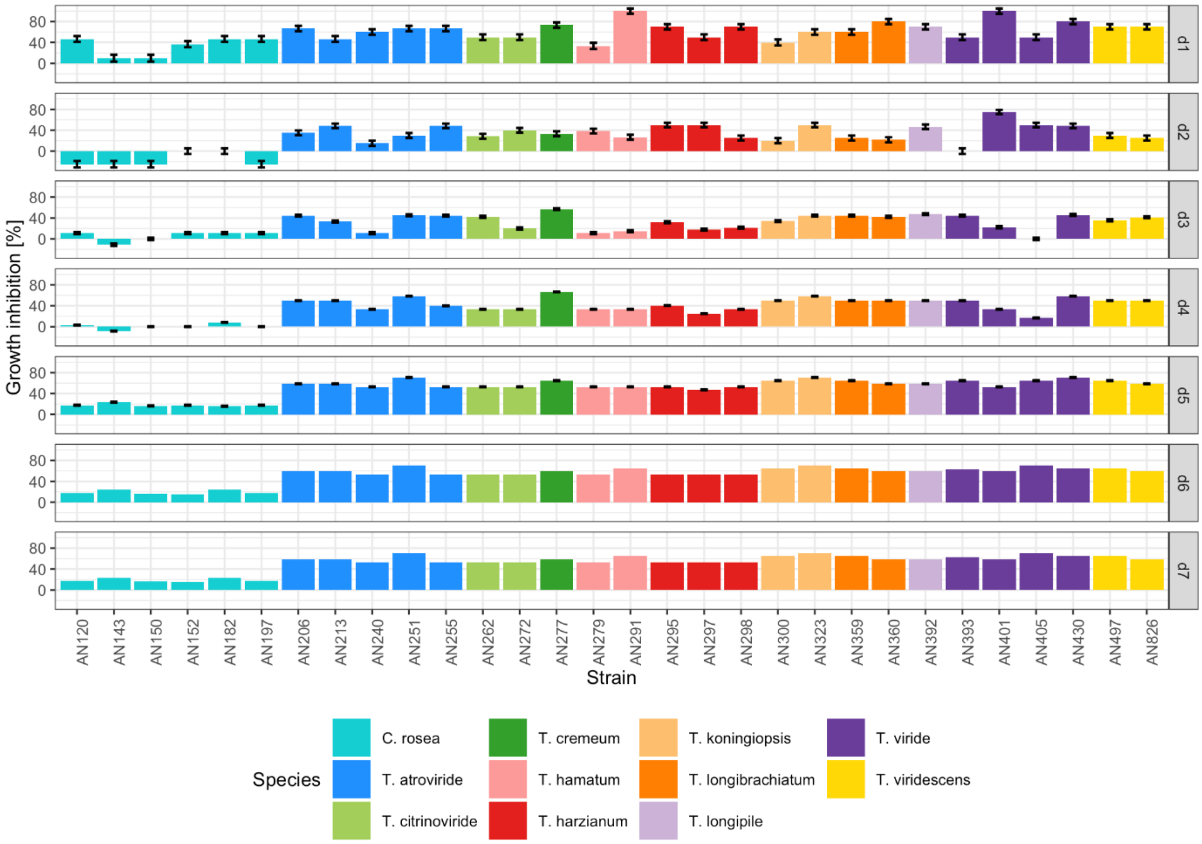

Table S3. The qualitative evaluation of the *F. culmorum* KF846 – *Clonostachys* and F. *culmorum* KF846 – *Trichoderma* interaction after 14 days of co-incubation on PDA medium by using modified Mańka [35].

	d1		d2		d3		d4		d5		d6		d7	
**Antagonist**	**d1.mean**	**d1.s.e.**	**d2.mean**	**d2.s.e.**	**d3.mean**	**d3.s.e.**	**d4.mean**	**d4.s.e.**	**d5.mean**	**d5.s.e.**	**d6.mean**	**d6.s.e.**	**d7.mean**	**d7.s.e.**
AN272	46.67	5.55	-25.00	6.16	11.11	2.11	2.78	0.71	17.65	0.91	17.65	NA	17.65	NA
AN291	10.00	6.59	-25.00	6.16	-11.11	2.36	-8.33	0.75	23.53	0.89	23.53	NA	23.53	NA
AN295	10.00	6.59	-25.00	6.16	0.00	2.23	0.00	0.72	16.47	0.92	16.47	NA	16.47	NA
AN297	36.67	5.80	0.00	5.44	11.11	2.11	0.00	0.72	17.65	0.91	15.29	NA	15.29	NA
AN298	46.67	5.55	0.00	5.44	11.11	2.11	8.33	0.69	15.69	0.92	23.53	NA	23.53	NA
AN300	46.67	5.55	-25.00	6.16	11.11	2.11	0.00	0.72	17.65	0.91	17.65	NA	17.65	NA
AN152	66.67	5.17	35.00	4.59	44.44	1.81	50.00	0.57	58.82	0.76	58.82	NA	58.82	NA
AN182	46.67	5.55	48.33	4.33	33.33	1.90	50.00	0.57	58.82	0.76	58.82	NA	58.82	NA
AN206	60.00	5.28	15.00	5.05	11.11	2.11	33.33	0.61	52.94	0.78	52.94	NA	52.94	NA

	d1		d2		d3		d4		d5		d6		d7	
**Antagonist**	**d1.mean**	**d1.s.e.**	**d2.mean**	**d2.s.e.**	**d3.mean**	**d3.s.e.**	**d4.mean**	**d4.s.e.**	**d5.mean**	**d5.s.e.**	**d6.mean**	**d6.s.e.**	**d7.mean**	**d7.s.e.**
AN240	66.67	5.17	30.00	4.69	45.19	1.80	58.33	0.55	70.59	0.73	70.59	NA	70.59	NA
AN497	66.67	5.17	48.33	4.33	44.44	1.81	40.00	0.59	52.94	0.78	52.94	NA	52.94	NA
AN262	50.00	5.48	28.33	4.73	42.22	1.82	33.33	0.61	52.94	0.78	52.94	NA	52.94	NA
AN393	50.00	5.48	40.00	4.48	20.00	2.02	33.33	0.61	52.94	0.78	52.94	NA	52.94	NA
AN392	73.33	5.07	33.33	4.62	57.04	1.72	66.67	0.54	64.71	0.75	58.82	NA	58.82	NA
AN120	33.33	5.89	38.33	4.52	11.11	2.11	33.33	0.61	52.94	0.78	52.94	NA	52.94	NA
AN277	100.00	4.90	26.67	4.77	14.81	2.08	33.33	0.61	52.94	0.78	64.71	NA	64.71	NA
AN150	70.00	5.12	50.00	4.30	31.85	1.91	40.56	0.59	52.94	0.78	52.94	NA	52.94	NA
AN279	50.00	5.48	50.00	4.30	17.78	2.04	25.00	0.64	47.06	0.80	52.94	NA	52.94	NA
AN360	70.00	5.12	25.00	4.81	21.48	2.01	33.33	0.61	52.94	0.78	52.94	NA	52.94	NA
AN143	40.00	5.71	20.00	4.92	34.07	1.89	50.00	0.57	64.71	0.75	64.71	NA	64.71	NA
AN251	60.00	5.28	50.00	4.30	44.44	1.81	58.33	0.55	70.59	0.73	70.59	NA	70.59	NA
AN197	60.00	5.28	25.00	4.81	44.44	1.81	50.00	0.57	64.71	0.75	64.71	NA	64.71	NA
AN213	80.00	5.00	21.67	4.88	42.22	1.82	50.00	0.57	58.82	0.76	58.82	NA	58.82	NA
AN359	70.00	5.12	46.67	4.36	47.41	1.78	50.00	0.57	58.82	0.76	58.82	NA	58.82	NA
AN255	50.00	5.48	0.00	5.44	44.44	1.81	50.00	0.57	64.71	0.75	62.35	NA	62.35	NA
AN401	100.00	4.90	75.00	3.96	22.22	2.00	33.33	0.61	52.94	0.78	58.82	NA	58.82	NA
AN430	50.00	5.48	50.00	4.30	0.00	2.23	16.67	0.66	64.71	0.75	70.59	NA	70.59	NA
AN826	80.00	5.00	48.33	4.33	45.93	1.80	58.33	0.55	70.59	0.73	64.71	NA	64.71	NA
AN323	70.00	5.12	30.00	4.69	35.56	1.88	50.00	0.57	64.71	0.75	64.71	NA	64.71	NA
AN405	70.00	5.12	25.00	4.81	41.48	1.83	50.00	0.57	58.82	0.76	58.82	NA	58.82	NA

Table S4. In-vitro toxin reduction in solid rice-medium (precalculated earlier in Genstat).

**pathogen**	**antagonist**	**% toxin reduction** (100% is complete reduction)						
		**3-AcDON**	s.e.	**DON**	s.e.	**ZEA**	s.e.	**avg**
KF846	AN240	99.57	1.57	97.19	5.27	99.04	4.44	KF846
	AN251	92.75	1.57	88.07	5.31	90.85	4.46	KF846
	AN291	100.00	1.57	96.89	5.27	99.83	4.44	KF846
	AN298	97.25	1.57	91.36	5.29	95.14	4.45	KF846
	AN430	99.13	1.57	93.44	5.28	94.77	4.45	KF846

Table S5. Measurements of toxin reduction in liquid solution of 3 selected antagonists + controls. The plots show again the means of the measurements +- sem.

**time**	**antagonist**	**3AcDON_mean**	**3AcDON_sem**	**DON_mean**	**DON_sem**	**ZEN_mean**	**ZEN_sem**
0	H2O	0.50	0.00	7.89	0.00	0.53	0.00
0	Medium	0.47	0.01	8.00	0.00	0.50	0.00
0	AN240	0.50	0.00	8.06	0.02	0.50	0.00
0	AN291	0.50	0.00	8.02	0.01	0.52	0.00
0	AN430	0.50	0.00	7.96	0.02	0.50	0.00
1	H2O	0.50	0.00	7.89	0.00	0.53	0.00
1	Medium	0.47	0.01	8.00	0.00	0.50	0.00
1	AN240	0.47	0.00	7.71	0.02	0.45	0.00
1	AN291	0.32	0.00	7.35	0.01	0.40	0.00
1	AN430	0.44	0.00	7.78	0.02	0.32	0.00
2	H2O	0.50	0.00	7.89	0.00	0.53	0.00
2	Medium	0.47	0.01	8.00	0.00	0.50	0.00
2	AN240	0.47	0.00	7.67	0.02	0.37	0.00
2	AN291	0.31	0.00	7.32	0.00	0.15	0.00
2	AN430	0.44	0.00	7.66	0.00	0.30	0.00
4	H2O	0.50	0.00	7.89	0.00	0.53	0.00
4	Medium	0.47	0.01	8.00	0.00	0.50	0.00
4	AN240	0.46	0.00	7.53	0.02	0.31	0.00
4	AN291	0.17	0.00	7.19	0.00	0.01	0.00
4	AN430	0.44	0.00	7.61	0.00	0.29	0.00
8	H2O	0.50	0.00	7.89	0.00	0.53	0.00
8	Medium	0.47	0.01	8.00	0.00	0.50	0.00
8	AN240	0.46	0.00	7.45	0.01	0.29	0.00
8	AN291	0.16	0.00	7.17	0.00	0.00	0.00
8	AN430	0.43	0.00	7.53	0.00	0.25	0.00
24	H2O	0.50	0.00	7.89	0.00	0.53	0.00
24	Medium	0.47	0.01	8.00	0.00	0.50	0.00
24	AN240	0.46	0.00	7.41	0.00	0.18	0.00

24	AN291	0.08	0.01	6.27	0.20	0.00	0.00
24	AN430	0.43	0.00	7.53	0.00	0.16	0.00
48	H2O	0.50	0.00	7.89	0.00	0.53	0.00
48	Medium	0.47	0.01	8.00	0.00	0.50	0.00
48	AN240	0.45	0.00	7.12	0.00	0.12	0.00
48	AN291	0.07	0.00	5.24	0.02	0.00	0.00
48	AN430	0.42	0.00	7.14	0.01	0.11	0.00
96	H2O	0.50	0.00	7.89	0.00	0.53	0.00
96	Medium	0.47	0.01	8.00	0.00	0.50	0.00
96	AN240	0.41	0.00	7.03	0.01	0.02	0.00
96	AN291	0.04	0.01	3.39	0.12	0.00	0.00
96	AN430	0.42	0.00	7.09	0.00	0.10	0.01

Table S6. Estimated linear regression coefficients analysis

antagonist	3AcDON_time.coef	3AcDON_signif.level	DON_time.coef	DON_signif.level	ZEN_time.coef	ZEN_signif.level
H2O	0.00		0.00		0.00	
Medium	0.00	**	0.02		0.00	
AN240	-0.01	***	-0.09	***	-0.05	***
AN291	-0.06	***	-0.29	***	-0.07	***
AN430	-0.01	***	-0.08	***	-0.05	***

Table S7. Correlation of the parallel content of DON and 3-AcDON mycotoxins during the cultivation of the *C. rosea* AN291 strain in a liquid medium with the addition of 3-AcDON or DON.

main.toxin	antagonist	time	toxin	mean	sd	sem
3AcDON	AN291	0	3AcDON	0.52	0.01	0.00
3AcDON	AN291	0	DON	0.16	0.01	0.00
3AcDON	AN291	1	3AcDON	0.40	0.01	0.00
3AcDON	AN291	1	DON	0.22	0.01	0.00
3AcDON	AN291	2	3AcDON	0.31	0.01	0.00
3AcDON	AN291	2	DON	0.46	0.03	0.01
3AcDON	AN291	4	3AcDON	0.40	0.00	0.00
3AcDON	AN291	4	DON	0.41	0.06	0.02
3AcDON	AN291	8	3AcDON	0.28	0.01	0.00
3AcDON	AN291	8	DON	0.45	0.04	0.01
3AcDON	AN291	24	3AcDON	0.40	0.01	0.00
3AcDON	AN291	24	DON	0.49	0.05	0.02
3AcDON	AN291	48	3AcDON	0.39	0.01	0.00
3AcDON	AN291	48	DON	0.49	0.02	0.01
3AcDON	AN291	96	3AcDON	0.27	0.01	0.00
3AcDON	AN291	96	DON	0.26	0.05	0.02
DON	AN291	0	3AcDON	0.14	0.02	0.01
DON	AN291	0	DON	0.51	0.01	0.00
DON	AN291	1	3AcDON	0.16	0.03	0.01
DON	AN291	1	DON	0.50	0.00	0.00
DON	AN291	2	3AcDON	0.14	0.01	0.00
DON	AN291	2	DON	0.34	0.01	0.00
DON	AN291	4	3AcDON	0.16	0.01	0.00
DON	AN291	4	DON	0.50	0.01	0.00
DON	AN291	8	3AcDON	0.13	0.03	0.01
DON	AN291	8	DON	0.27	0.02	0.01
DON	AN291	24	3AcDON	0.16	0.02	0.01
DON	AN291	24	DON	0.48	0.01	0.00
DON	AN291	48	3AcDON	0.15	0.01	0.00
DON	AN291	48	DON	0.47	0.02	0.01
DON	AN291	96	3AcDON	0.11	0.01	0.00
DON	AN291	96	DON	0.25	0.01	0.00

Table S8. The differences in mass compared to the control (no or pathogens inoculation) groups are expressed as a relative % change of control values.

Cultivar	Season	Antagonist	Mass change [%]	Mass change s.e.
Legenda	I	AN240	7.52	9.89
		AN291	-2.46	9.41
		AN430	-1.07	9.47
	II	AN240	47.43	20.66
		AN291	34.03	19.40
		AN430	63.65	22.25
Bombona	I	AN240	4.31	11.07
		AN291	17.64	11.83
		AN430	9.76	11.38
	II	AN240	26.79	10.77
		AN291	25.43	10.70
		AN430	30.14	10.95

Table S9. The differences in toxins content to the control (inoculation by pathogen only) are expressed as a % reduction of toxins compared to the control.

Cultivar	season	antagonist	3AcDON_est	3AcDON_se	DON_est	DON_se	ZEN_est	ZEN_se
Bombona	I	AN240	38.10	18.26	14.54	2.41	46.73	1.50
	I	AN291	92.86	15.56	11.83	2.44	6.03	1.80
	I	AN430	28.57	19.08	41.23	2.12	9.05	1.70
	II	AN240	71.76	5.17	16.19	5.49	29.27	7.60
	II	AN291	64.12	5.29	27.35	5.20	29.27	7.60
	II	AN430	58.78	5.38	8.31	5.71	26.83	7.70
Legenda	I	AN240	71.43	27.40	72.73	4.16	24.24	9.75
	I	AN291	80.00	26.90	52.53	4.44	13.64	10.27
	I	AN430	100.00	26.40	56.81	4.37	40.91	9.03
	II	AN240	53.19	14.47	38.43	5.28	44.44	6.52
	II	AN291	100.00	13.10	40.11	5.24	28.89	6.99
	II	AN430	48.94	14.72	27.55	5.55	42.22	6.58

Table S10. Biomass of *F. culmorum* determined by qPCR in culture/ co-culture on rice solid medium and in wheat kernels (Bombona, Legenda) inoculated with the pathogen (KF846, control) and inoculated with both the pathogen (KF846) and the antagonist (AN240 or AN291 or AN430).

Treatment	Experiment	Substrate	mean	sem	Letters.sep
KF846 - control	semi-field experiment	Bombona kernels	447.33	32.20	a
KF846+AN240			220.33	23.76	b
KF846+AN291			57.00	33.15	c
KF846+AN430			210.33	5.21	b
KF846 - control		Legenda kernels	634.00	9.60	a
KF846+AN240			295.67	1.46	b
KF846+AN291			123.00	1.53	c
KF846+AN430			327.00	9.17	b
KF846 - control	solid substrate bioassay	autoclaved rice	837.21	7.51	a
KF846+AN240			405.19	6.94	b
KF846+AN291			210.72	6.81	c
KF846+AN430			488.80	15.01	b

*Letters: common characters identify levels or groups that are not significantly different, based on results of TukeyHSD test at alpha=0.05 for each trait.

Table S11. Comparison of TKW and HI in control wheat plants and after *Trichoderma* root inoculation.

cultivar	treatment	TKW_mean	TKW_sem	TKW_Letters	HI_mean	HI_sem	HI_Letters
Bombona	Control	40.29	0.19	d	0.36	0.00	a
Bombona	AN35	36.52	0.19	e	0.34	0.00	ab
Bombona	AN392	42.51	0.40	bc	0.34	0.00	b
Legenda	Control	42.82	0.22	b	0.24	0.00	c
Legenda	AN35	41.50	0.18	c	0.15	0.00	e
Legenda	AN392	47.94	0.22	a	0.21	0.01	d

*Letters: common characters identify levels or groups that are not significantly different, based on results of TukeyHSD test at alpha=0.05 for each trait.

Table S12. Toxin content in wheat kernels and chaff after *Trichoderma* root inoculation.

Cultivar	Treatment	Part.of.an.ear	ZEN_mean	ZEN_sem		NIV_mean	NIV_sem		EnB_mean	EnB_sem		EnB1_mean	EnB1_sem	
Bombona	control	grain	16.36	0.16	e	1880.14	11.69	ef	5923.62	18.71	c	3729.73	23.11	c
Bombona	control	grain chaff	17.39	0.33	e	4457.14	97.17	a	0.00	0.00	g	0.00	0.00	g
Bombona	AN35	grain	43.17	2.47	a	1680.08	6.78	f	2525.71	28.06	f	1795.47	11.66	f
Bombona	AN35	grain chaff	22.62	0.62	d	3321.62	27.06	b	0.00	0.00	g	0.00	0.00	g
Bombona	AN392	grain	0.00	0.00	g	2312.55	51.52	d	4214.83	59.85	d	5811.45	11.83	a
Bombona	AN392	grain chaff	34.01	0.65	b	3134.82	91.61	b	0.00	0.00	g	0.00	0.00	g
Legenda	control	grain	0.00	0.00	g	1743.28	5.33	f	7139.38	12.09	a	2846.68	49.24	d
Legenda	control	grain chaff	0.00	0.00	g	0.00	0.00	g	0.00	0.00	g	0.00	0.00	g
Legenda	AN35	grain	0.00	0.00	g	2475.91	38.60	cd	6660.90	24.61	b	4654.08	17.87	b
Legenda	AN35	grain chaff	6.35	0.32	f	2598.12	7.99	c	0.00	0.00	g	0.00	0.00	g
Legenda	AN392	grain	0.00	0.00	g	0.00	0.00	g	3543.63	34.30	e	2169.50	2.67	e
Legenda	AN392	grain chaff	28.97	0.40	c	2057.59	38.90	e	0.00	0.00	g	0.00	0.00	g
